# Suspected Post-intubation Onset of Lemierre Syndrome: A Case Report

**DOI:** 10.7759/cureus.40901

**Published:** 2023-06-24

**Authors:** Nicole F Grigoryants, Danielle Glinka, Fulton Defour

**Affiliations:** 1 Medicine, Alabama College of Osteopathic Medicine, Mobile, USA; 2 Medicine, Alabama College of Osteopathic Medicine, Dothan, USA; 3 Internal Medicine, Thomas Hospital, Fairhope, USA

**Keywords:** septic thrombophlebitis, thrombosis of the internal jugular vein, intubation complication, acute pharyngitis, lateral neck mass, internal jugular vein thrombophlebitis, lemierre's syn

## Abstract

Lemierre syndrome (LS) is an infectious thrombophlebitis of the internal jugular vein (IJV) that typically occurs in previously healthy, young individuals after a recent oropharyngeal bacterial illness. Here, we present the case of a 63-year-old female who presented six days after trauma to the oropharynx from intubation for lumbar interbody fusion with fever, dysphagia, and pain and swelling of the neck. Imaging confirmed IJV thrombosis spanning C2 to C5; however, blood cultures were negative on two separate occasions. Treatment with IV antibiotics led to rapid clinical improvement compared to baseline.

## Introduction

Lemierre syndrome (LS) is a rare complication of a bacterial oropharyngeal infection that typically presents in previously healthy young adults. This oropharyngeal infection and abscess formation result in inflammation of nearby structures, resulting in complications including thrombosis of the internal jugular vein (IJV), septic embolization from abscess drainage, and bacteremia [1]. The exact parameters of LS have been widely variable; some sources define this disease process as one specifically due to infection by Fusobacterium necrophorum, a Gram-negative nonmotile anaerobe; some sources endorse a definition involving infection by any anaerobe; and others do not include a bacterial infection due to any pathogen whatsoever in their definition of the condition [2]. One such case of the last-mentioned was that of a 25-year-old male who developed LS status post-blunt-force neck trauma due to a motor vehicle accident [3]. The pathophysiology of LS in the context of F. necrophorum remains unclear, but some sources speculate hematogenous spread via the tonsillar vein [4]. Some sources require positive blood cultures; some sources do not require IJV thrombosis whatsoever [5]. This may prove a challenge in the proper diagnosis of LS, as no consensus has been reached. Primary infection varies widely, ranging from infection of the tonsils or peritonsillar tissue to infection of the mastoid bone, middle ear, teeth, or gingiva [6]. Finally, the rarity of this disease limits clinical evidence in the pathophysiology, clinical presentation and progression, prognosis, and management of LS.

## Case presentation

History 

A 63-year-old female with a history of degenerative disc disease, status post-L4-L5 interbody fusion with cage six days ago, presents to the emergency department with a chief complaint of pain and swelling in her right lateral neck worsening over the past five days. The pain is worse with movement and swallowing and radiates to the right ear. An additional review of systems was positive for subjective fever, chills, sore throat, and diaphoresis. The patient denied congestion and voice changes.

Physical exam

The patient presented to the ED alert, afebrile, and normotensive without acute distress. The physical exam was notable for a hard, tender 5-cm longitudinal mass found along the anterior margin of the sternocleidomastoid muscle. The mass was not fluctuant, erythematous, or pulsatile. No carotid bruits were detected upon examination. The oropharynx was without erythema or swelling, with moist mucous membranes. There was no cervical lymphadenopathy noted.

Diagnostic testing

**Figure 1:**
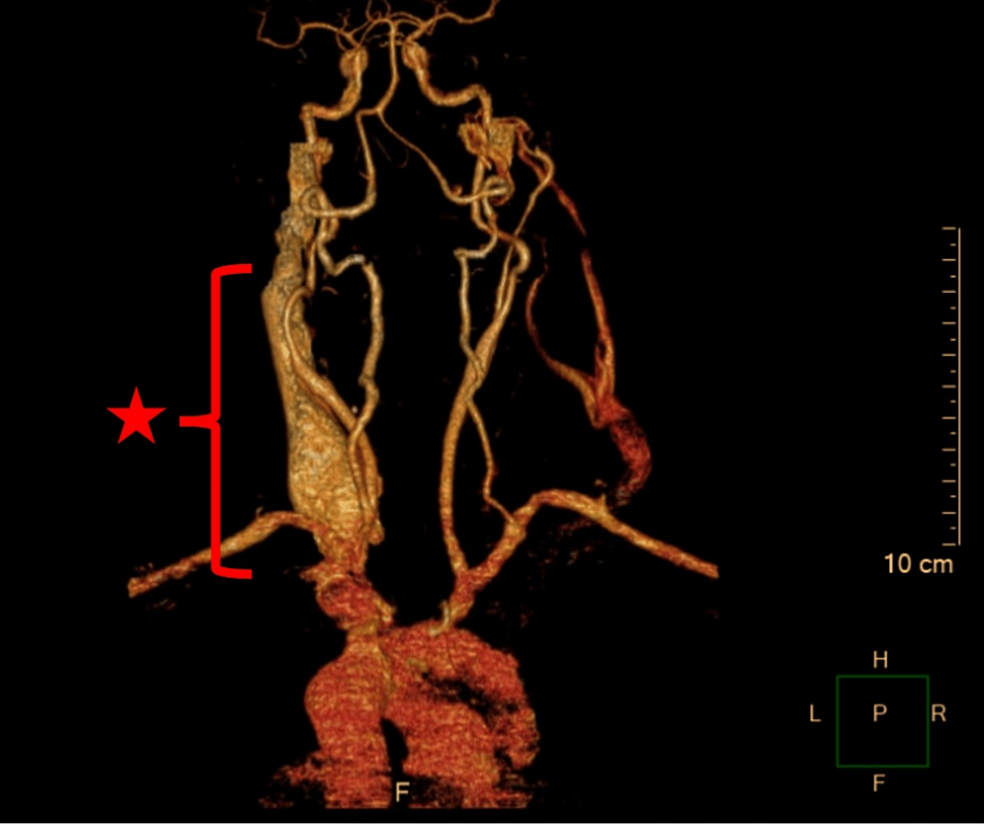
Computed tomography angiography of the neck showing irregular appearance of the right internal jugular in a 63-year old female This figure shows diffuse asymmetric enlargement and non-opacification of the right internal jugular vein (IJV) with adjacent soft tissue stranding (red bracket). Note that there is no appreciable right carotid stenosis or thrombosis, further excluding other diagnoses. These results are compatible with septic thrombophlebitis of the right IJV.

Results of a complete blood count showed leukocytosis (16.1 K/µl) with 84% neutrophils, thrombocytosis, and microcytic anemia. Electrolytes were found to be within normal limits; however, mild albuminemia was found. Blood cultures, done on hospitalization day 2, found no growth, and throat cultures, collected on hospitalization day 3, found only normal respiratory flora. In order to determine if an abscess was present, computed tomography (CT) with intravenous (IV) contrast was performed and found a significantly enlarged right IJV with thrombosis spanning from the level of C2 to C5, as well as mild cervical lymphadenopathy. Together, these findings suggested septic thrombophlebitis of the right internal jugular vein. This thrombosis also appeared to be the source of the mass effect, causing a midline shift to the left; however, left-sided venous structures were found to be within normal limits and did not display signs of appreciable overcompensation. Phlegmonous changes were noted at the right internal carotid artery, which prompted further investigation with computed tomography angiography (CTA) of the neck with IV contrast. This CTA showed no suspicious abnormalities of the right internal carotid artery and confirmed previous findings on CT. Figure 1 shows the irregular appearance of the right internal jugular vein, compatible with septic thrombophlebitis.

Treatment course

The patient was started on empiric antibiotic treatment with IV piperacillin/tazobactam and vancomycin. Following consultation with infectious disease specialists, piperacillin/tazobactam was discontinued to be replaced by ampicillin/sulbactam (3 g/100 ml NS) four times daily. An IV dose of alteplase was used for anticoagulation. A peripherally inserted central catheter (PICC) was placed on hospitalization day 6 for the continuation of antibiotic treatment in the outpatient setting for 22 days. The patient steadily improved clinically during her hospital course, which was reflected in laboratory findings with a return to a normal leukocyte count. She was discharged home on hospitalization day 7. At the patient’s outpatient one-week follow-up, she reported a clinical return to baseline despite some mild residual swelling in the neck without erythema or tenderness, as well as a radiologic return to baseline, which was noted on a chest radiograph.

## Discussion

Epidemiology and pathogenesis

LS is a rare condition in our current post-antibiotic era, characterized as superficial thrombophlebitis of the IJV. The incidence of LS is estimated by some studies to be around three cases per one million people, though this number was much greater before the widespread use of antibiotics for Streptococcal pharyngitis became commonplace [7]. Generally, LS is most commonly found in previously healthy children or young adults, most often within one to three weeks after a throat infection or case of pharyngitis [7]. The mode of transmission is unclear, with some studies considering the possibilities of both animal-to-human and human-to-human sources [2]. This lack of consensus on the precise definition of LS may pose a challenge for clinicians today, but it is altogether nuanced since complications, treatments, and outcomes do not differ between LS and other suppurative thrombophlebitides of the neck.

Clinical presentation and diagnosis

The primary difficulty in diagnosing LS is a lack of clinical criteria, which is a result of an unclear description of the disease. Therefore, there is an enormous variety in clinical presentations. However, typical manifestations include fever, sore throat, neck pain, exudative tonsillitis, and respiratory symptoms such as dyspnea or pleurisy [5]. Rigors and chills were a symptom specifically highlighted by André Lemierre, with onset anywhere from 4 to 12 days after that of the sore throat [2]. Swelling or induration of the neck, jaw, or sternocleidomastoid muscle may or may not be present. A review done by Chirinos et al. spanning 109 cases of LS found that up to 47% of patients did not have any significant manifestations of thrombosis in the neck whatsoever [8]. More rarely, reports have noted Horner syndrome and Horner syndrome-like cranial nerve palsies accompanying the IJV thrombosis [2].

The diagnosis may be confirmed with imaging demonstrating IJV thrombosis or a filling defect. Radiographic imaging may also further reveal pulmonary metastases, such as empyema or effusion, abscess, or pneumothorax [5]. Blood cultures may or may not be confirmatory, as bacteremia may be transient [5]. In the instance of metastasis, fluid may be drawn from these secondary sites of infection to isolate the pathogen of interest as well. Elevated liver enzymes associated with hyperbilirubinemia have also been linked to LS [8].

Treatment and prognosis

LS treatment revolves around antimicrobial therapy with activity against F. necrophorum and oral streptococci. Treatments include piperacillin-tazobactam, ampicillin-sulbactam, imipenem or meropenem, or ceftriaxone plus metronidazole. Antimicrobial therapy is largely dependent on clinical circumstances, but typically four weeks of antibiotics with at least two weeks of IV therapy are recommended [5]. While LS is life-threatening, the mortality rate is reported to be between 5% and 18%, with septic emboli and end-organ damage affecting long-term morbidity [9].

Limitations

It is difficult to discern if there was any pre-existing underlying pharyngitis that was concomitant with the surgical operation. While the patient did not begin experiencing symptoms of throat pain and dysphagia until after her operation, the possibility that she began experiencing symptoms of previously acquired bacterial pharyngitis on postoperative day 1 must also be considered. Our current working theory is that this patient’s LS was a result of the introduction of oropharyngeal microbiota after intubation. However, we cannot rule out the interbody fusion operation as a possible source of infection. Additionally, the negative blood cultures on two separate occasions also warrant further discussion. While bacteremia can be transient and some sources do not require positive blood cultures for a diagnosis of LS, we consider a limitation in this study to be the inability to pinpoint the exact microbiological etiology of this case. Perioperative antibiotics may have played a factor in the negative cultures, but it is impossible to definitively determine the etiology of this case of LS. Regardless, the patient responded positively to the ampicillin/sulbactam regimen.

## Conclusions

The purpose of this case study was to highlight a possible presentation of Lemierre syndrome in a previously healthy 63-year-old female after lumbar interbody fusion. While LS typically presents in younger patients with recent presentations of bacterial pharyngitis, there have been previously documented instances of IJV thrombosis as a result of blunt-force trauma. Overall, it is important to note that LS may be widely underestimated due to a lack of awareness surrounding the disease. The case further highlights the need to consider Lemierre syndrome in the presentation of a unilateral neck mass and signs of infectious pharyngitis post-intubation.
